# Bispecific Antibody Armed Metabolically Enhanced Headless CAR T Cells

**DOI:** 10.3389/fimmu.2021.690437

**Published:** 2021-07-05

**Authors:** Archana Thakur, John Scholler, Ewa Kubicka, Edwin T. Bliemeister, Dana L. Schalk, Carl H. June, Lawrence G. Lum

**Affiliations:** ^1^ Department of Medicine, Division of Hematology/Oncology, University of Virginia, Charlottesville, VA, United States; ^2^ Center for Cellular Immunotherapies, University of Pennsylvania, Philadelphia, PA, United States

**Keywords:** breast cancer, pancreatic cancer, activated T cells, co-activated T cells, bispecific antibody, headless CAR T cells, Th_1_ cytokines

## Abstract

Adoptive T cell therapies for solid tumors is challenging. We generated metabolically enhanced co-activated-T cells by transducing intracellular co-stimulatory (41BB, ICOS or ICOS-27) and CD3ζ T cell receptor signaling domains followed by arming with bispecific antibodies (BiAbs) to produce armed “Headless CAR T cells” (hCART). Various hCART armed with BiAb directed at CD3ϵ and various tumor associated antigens were tested for: 1) specific cytotoxicity against solid tumors targets; 2) repeated and dual sequential cytotoxicity; 3) survival and cytotoxicity under *in vitro* hypoxic condition; and 4) cytokine secretion. The 41BBζ transduced hCART (hCART_41BBζ_) armed with HER2 BiAb (HER2 hCART_41BBζ_) or armed with EGFR BiAb (EGFR hCART_41BBζ_) killed multiple tumor lines significantly better than control T cells and secreted Th_1_ cytokines/chemokines upon tumor engagement at effector to target ratio (E:T) of 2:1 or 1:1. HER2 hCART serially killed tumor targets up to 14 days. Sequential targeting of EGFR or HER2 positive tumors with HER2 hCART_41BBζ_ followed by EGFR hCART_41BBζ_ showed significantly increased cytotoxicity compared single antigen targeting and continue to kill under *in vitro* hypoxic conditions. In summary, metabolically enhanced headless CAR T cells are effective serial killers of tumor targets, secrete cytokines and chemokines, and continue to kill under *in vitro* hypoxic condition.

## Introduction

Chimeric antigen receptor (CAR) T cells (CAR-T) immunotherapy has been shown to be clinically effective in inducing lasting remissions in hematological malignancies (1–3), however, early phase clinical trials in solid tumors using CAR-T have met with limited success (4–8). The major challenges of CAR-T in solid tumors have been the lack of efficacy, T cell exhaustion, off-tumor toxicity, cytokine release syndrome (CRS), and metabolic insufficiency of CAR-T to persist and provide effector functions in the immunosuppressive tumor microenvironment (TME) (9–14). To address these challenges, we combined the potent intracellular signaling domains of CAR-T with the bispecific antibody (BiAb) arming strategy to redirect the non-MHC restricted cytotoxicity of co-activated T cells. The genetically engineered signaling domains of “Headless CAR-T cells” (hCART) contain the transmembrane, the intracellular domain (ICD) of the co-stimulatory receptors, and the T cell receptor signaling-CD3ζ domains of a CAR-T except the extracellular scFv CAR domain.

The rationale for combining hCART with the BiAb arming approach is based on *in vitro* and *in vivo* clinical evidence that shows: 1) Bispecific antibody Armed T cells (BATs) have shown encouraging clinical results in breast (15, 16), prostate (17), and pancreatic cancer (18); 2) adapting the BATs platform strategy for arming hCART permits choice of any BiAb for redirecting specific cytotoxicity; 3) BATs release cytokines/chemokines upon tumor engagement and block the suppressive properties of myeloid-derived suppressor cell (MDSC) and T regulatory cells (T_REGs_) in the TME (19, 20). Infusions of BATs armed with anti-CD3 x anti-HER2 BiAb (HER2 BATs) in metastatic breast cancer patients were safe without CRS and induced endogenous cellular and humoral immune responses that persisted up to 4 months post infusion (15).

This flexible BiAb arming approach will facilitate the adjustment of arming dose of BiAb and dose and frequency of armed hCART infusions. In addition, BiAb loaded on hCART would be diluted with each cell division and may provide a self-braking mechanism on the hCART to decrease the risk of CRS seen in CAR-T therapies. This study addresses the following questions: 1) Can the adaptable BiAb armed hCART engineering platform target different tumor antigens? 2) Can BiAb armed hCART target multiple tumor associated antigens? 3) Will BiAb armed hCART remain functionally active under an *in vitro* hypoxic environment? 4) Can BiAb armed hCART proliferate and mediate serial killing of tumors?

This study shows that hCART transduced with 41BBζ (BTC_41BBζ_) armed with BiAbs are superior to non-genetically modified T cells armed with BiAbs (BATs) and mediate superior levels of cytotoxicity directed at all tested solid tumor lines as well as kill in a hypoxic environment.

## Materials and Methods

### T Cell Activation and Expansion

Activated T cells (ATC) were generated from peripheral blood mononuclear cells (PBMCs) by activation with 20 ng/million cells of anti-CD3 monoclonal antibody (OKT3). ATC were expanded by adding 100 IU/million cells of IL-2 every other day for 14 days in RPMI-1640 supplemented with 10% FBS. Harvested ATC were armed with bispecific antibody anti-CD3 x anti-EGFR [EGFRBi] or anti-CD3 x anti-HER2 [HER2Bi] at a pre-optimized concentration of 50 ng/10^6^ ATC. Similar to ATC, co-activated T cells (COATC) were generated from PBMCs by activating T cells with anti-CD3/anti-CD28 beads (Dynabeads, Thermofisher) at the 3:1 bead:cell ratio and transduced with lentivirus (LV) at day 2 (multiplicity of infection, MOI: 5). Beads were removed by magnetic separation at day 6 and cells were expanded till day 14 by adding 100 IU/million cells of IL-2 every other day. Harvested COATC were armed with EGFRBi or HER2Bi at pre optimized concentration of 50 ng/10^6^ COATC.

### Design of “Headless” CAR Construct and Production of Headless CAR T Cells (hCART)

**Figure 1:**
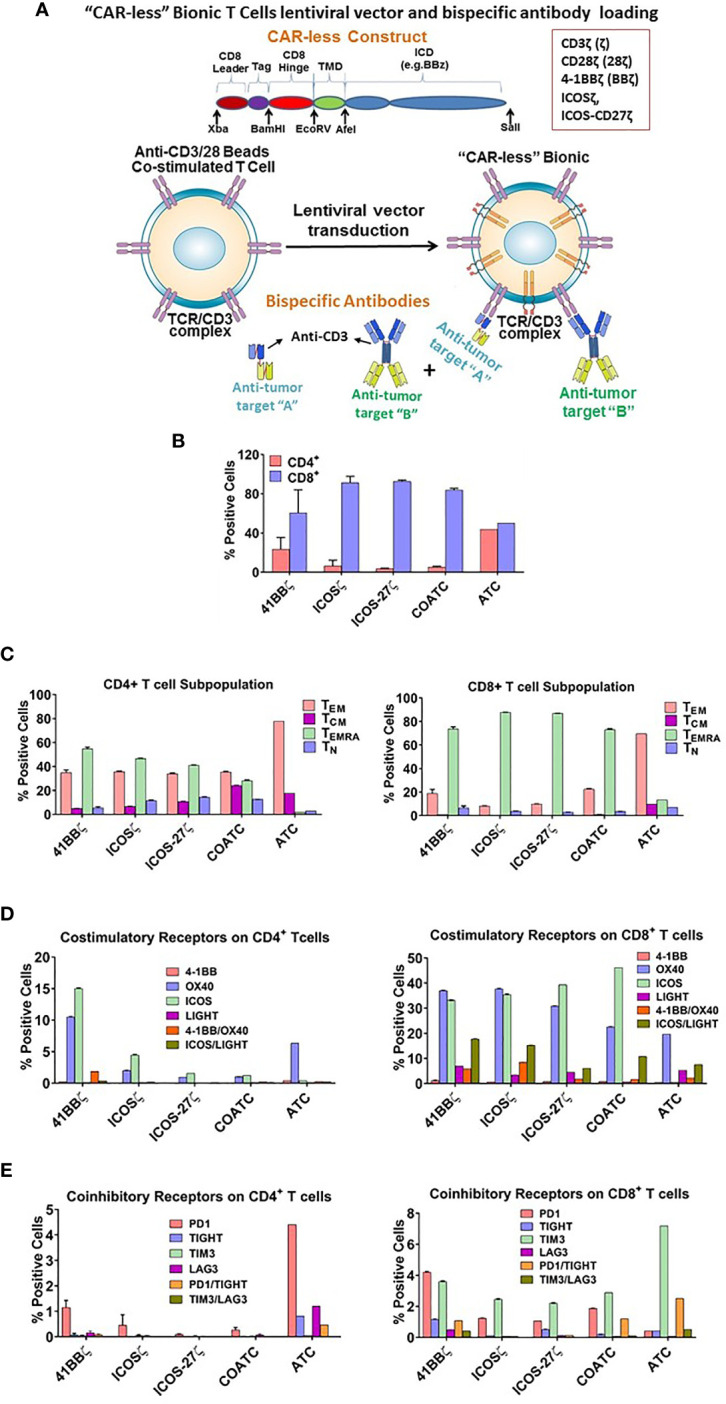
**(A)** Vector Construct and Bispecific Antibody Loading of Headless CAR T cells. Shows a sequence and basic structure of an intracellular signaling domain of the construct. The intracellular signaling domain includes a fusion protein with a detectable tag, hinge/transmembrane domain, and intracellular domain inserted in lentivirus vector. T cells are co-activated with anti-CD3/anti-CD28 antibody coated beads, transduced with lentivirus vector and expanded for 10-14 days in IL-2. Co-activated T cells (COATC) containing the intracellular signaling domain are defined as “Headless CAR T cells” (hCART) and targeting ability of hCART is facilitated by loading or arming them with bispecific antibodies (BiAb) that are produced either by chemical heteroconjugation of whole IgG molecules with Fc-Fc permanent covalent linker or by recombinant technology. hCART can be armed with single, dual, or triple loading with one, two, or three different BiAbs to target multiple tumor antigens. **(B)** T cell Subpopulations and Stimulatory/Inhibitory Co-Receptor Expression on hCART. **(B)** shows the CD4^+^/CD8^+^ T cell ratios of hCART_41BBζ_, hCART_ICOSζ_, hCART_ICOS-CD27ζ_, COATC and ATC after one month in culture (n=3). **(C)** Shows the percent positive CD4^+^ and CD8^+^ T cell subpopulations of hCART_41BBζ_, hCART_ICOSζ_, hCART_ICOS-CD27ζ_, COATC and ATC (n=3). **(D)** Shows the expression pattern of co-stimulatory receptors on hCART_41BBζ_, hCART_ICOSζ_, hCART_ICOS-CD27ζ_, COATC and ATC after long term culture [1 month; (n=3)]. **(E)** Shows the expression of co-inhibitory receptors of hCART_41BBζ_, hCART_ICOSζ_, hCART_ICOS-CD27ζ_, COATC and ATC on CD4^+^ and CD8^+^ cells derived from the same donors (n=3).

Various combinations of second and third generation self-inactivating lentiviral vector (LV) expressing CAR-less TCR signal-transduction domain CD3 zeta (ζ) alone or in combination with co-stimulatory molecules were generated as shown in **Figure 1A**. The intracellular co-stimulatory signaling domains include CD28ζ, 41BBζ, ICOSζ, and ICOS-CD27ζ constructed in tandem were selected to enhance the metabolic function of hCART. **Table 1** shows the effector cells, their respective acronyms, type of stimulation, the ICD construct in the lentiviral vector, and the BiAb used to engineer the various effector populations. The effector populations evaluated in this study can be divided into 2 groups: 1) anti-CD3 activated T cells (ATC) or anti-CD3/anti-CD28 co-activated T cells (COATC) armed with HER2Bi or EGFRBi; 2) Headless CAR T cells (hCART) are COATC transduced with different CAR-less constructs [CD28ζ, 41BBζ, ICOSζ and ICOS-CD27ζ] e.g. hCART transduced with 41BBζ and armed with HER2Bi are referred as HER2 hCART_41BBζ_ or armed with EGFRBi referred as EGFR hCART_41BBζ_. The LV construct containing CD8 leader, Tag, CD8 Hinge, transmembrane domain, and ICD is shown in **Figure 1A**.

**Table 1:** Shows the effector cells, their respective acronyms, type of stimulation, the ICD construct in the lentiviral vector, and the arming BiAb.

Term	Effector	Stimulation	Vector Transduced	Arming BiAb
Coactivated T cell (COATC)	COATC	CD3/CD28	None	Unarmed
HER2Bi Armed COATC	HER2 COATC	CD3/CD28	None	HER2Bi
EGFRBi Armed COATC	EGFR COATC	CD3/CD28	None	EGFRBi
Activated T cell (ATC)	ATC	anti-CD3	None	Unarmed
HER2Bi Armed ATC	HER2 BATs	anti-CD3	None	HER2Bi
EGFRBi Armed ATC	EGFR BATs	anti-CD3	None	EGFRBi
Headless CAR T cells (hCART)	hCART	CD3/CD28	Yes	
hCART_41BBζ_	hCART	CD3/CD28	41BBζ	Unarmed
HER2 hCART_41BBζ_				HER2Bi
EGFR hCART_41BBζ_				EGFRBi
hCART_ICOSζ_	hCART	CD3/CD28	ICOSζ	Unarmed
HER2 hCART_ICOSζ_				HER2Bi
EGFR hCART_ICOSζ_				EGFRBi
hCART_ICOS-27ζ_	hCART	CD3/CD28	ICOS-27ζ	Unarmed
HER2 hCART_ICOS-27ζ_				HER2Bi
EGFR hCART_ICOS-27ζ_				EGFRBi
hCART_GFP_	hCART	CD3/CD28	GFP	Unarmed
HER2 hCART_GFP_				HER2Bi
EGFR hCART_GFP_				EGFRBi
hCART_CD28ζ_	hCART	CD3/CD28	CD28ζ	
hCART_ζ_	hCART	CD3/CD28	ζ	

### Production of BiAb

The BiAbs were produced via the chemical heteroconjugation of OKT3 and Erbitux (humanized anti-EGFR IgG_1_, ImClone LLC., Branchburg, NJ), OKT3 and Herceptin (a humanized anti-HER2 IgG_1_, Genentech Inc., South San Francisco, CA) or OKT3 and Rituxan (a humanized anti-CD20 IgG_1_, Genentech Inc., South San Francisco, CA) as described (21, 22).

### Arming of T Cells

The hCART, COATC and ATC were armed with OKT3 x anti-HER2 BiAb (HER2Bi) [HER2 hCART] or anti-OKT3 x anti-EGFR BiAb (EGFRBi) [EGFR hCART] using optimized arming concentration of 50 ng of HER2Bi or EGFRBi/million cells (21).

The human breast cancer cell lines (MDA-MB-231 [MB231], SK-BR-3 [SKBR3], MCF-7) were obtained from ATCC and were maintained in DMEM culture media (Lonza Inc., Allendale, NJ) supplemented with 10% FBS (Lonza Inc.), 2 mM L-glutamine (Invitrogen, Carlsbad, CA), 50 units/ml penicillin, and 50 µg/ml streptomycin (Invitrogen). These cell lines were used in experiments only up to 10 passages and after 10 passages a new original ATCC stock was used to initiate the cell line culture and cultured up to 3 passages before using them the experiments. Non-ATCC stocks were not used for the study.

### ^51^Chromium (^51^Cr) Release Assay for Specific Cytotoxicity

To target adherent tumor cell lines, target cells were plated in 96-well flat-bottom microtiter plates at 4x10^4^ cells/well, allowed to adhere overnight at 37°C, and labeled with ^51^Cr at 20 µCi/mL in the labeling media (50% fetal bovine serum (FBS) in complete RPMI-1640 medium supplemented with 10% FBS, 2% penicillin-streptomycin, and 1% L-glutamine as described (21). Effectors, unarmed or armed hCART, unarmed or armed COATC or unarmed or armed ATC were then added to achieve effector:target (E:T) ratios of 10:1. Co-cultures were incubated for 18 hours (adherent cell lines) and the supernatants were collected for liquid scintillation counting in order to quantitate the amount of ^51^Cr released. Percent specific cytotoxicity was calculated as follows: (experimental counts per minute (cpm) – spontaneous cpm)/(maximum cpm – spontaneous cpm) × 100. Means and standard errors were calculated from four to six replicates per sample.

### Real Time Cytotoxicity

In the Real Time Cell Analysis (RTCA) system, cytotoxicity is measured by cellular impedance readout as Cell Index (CI) to monitor real-time changes in cell number. Cell attachment was monitored using the RTCA software until the plateau phase was reached, which was usually after approximately 22-24 hours before adding effector cells as described previously (23). We used breast cancer cell lines (MB231, MCF-7, SKBR3) as targets (n=4, each condition had 3-4 replicates). The target cells (10-20,000 cells/well was optimal for each cell line) were plated in 96-well E-Plates, cells were allowed to adhere overnight or longer until reaching the CI of 1.0, followed by adding effectors (unarmed or armed hCART, unarmed or armed COATC or unarmed or armed ATC) at E:T of 2:1 or 1:1. For sequential killing, tumor cells were incubated with HER2 hCART for 24 hours followed by adding EGFR hCART or vice versa at 2:1 or 1:1 E:T ratios, target cells’ impedance signals were continuously monitored for 72-120 hours in 15 minute intervals. Untreated targets or effectors without targets served as controls. To analyze the acquired data, CI values were exported and percentage of lysis was calculated in relation to the tumor cell impedance without effector cells.

### Serial Killing Assay

Serial killing assays by armed and unarmed hCART, COATC, and ATC were performed as described previously (23). Briefly, armed and unarmed hCART, COATC and ATC effectors were co-cultured with MCF-7 breast cancer cell line (n=3, each condition had 4-6 replicates) at 2:1 E:T ratio for 72 hours (Initial co-culture). Effector cells from the initial co-cultures were serially transferred to new plates with new target cells for subsequent rounds of killing (2^nd^, 3^rd^, and 4^th^). With each transfer, effectors were counted and re-suspended in fresh media supplemented with IL-2 to adjust the E:T ratio. Long-term serial killing of MCF-7 by different type of effectors with without HER2Bi arming were sequentially monitored in a RTCA using xCELLigence.

### Effect of Hypoxia on the Functional Activities of Headless CAR T Cells

Before determining the effect of hypoxia on the functional activities of effector cells, we determined the effects of hypoxia on the survival of MCF-7 target cells and on effectors using hypoxia-mimetic Cobalt Chloride (CoCl_2_) by RTCA and flow cytometry, respectively. Tumor cells were plated at 10,000 cells/well in 96-well plates and incubated overnight at 37°C in 5% CO_2_. Once adherent cells reached a CI of 1.0, cells were treated with 0 µM - 400 µM concentrations of CoCl_2_ to induce hypoxia tracked by RTCA up to 120 hours and the effect of hypoxia on the T cell viability was performed using the same doses of CoCl_2_ by flow cytometry. A non-toxic CoCl_2_ 100 µM dose was used to determine the effect of hypoxia on the functional activity of hCART (n=3).

### Cytokines and Chemokines Profile of Target and Effector Co-Cultures

COATC, hCART, and BATs (n=2, each pooled from three replicates) either left unarmed or armed with HER2Bi or EGFRBi were co-cultured overnight in the absence or presence of targets followed by collection of the cell-free supernatants to quantitated multipanel (R&D Systems) 45 cytokines, chemokines and growth factors using a Bio-Plex 200 system (BIO-RAD, Hercules, CA). The values are reported in pg/ml of culture supernatants.

### Phenotyping for Memory, Co-Stimulatory and Co-Inhibitory Receptors on Headless CAR T Cells After Long-Term Expansion

We assessed the phenotype of hCART transduced with different constructs after 4 weeks of culture (n=3). Antibodies used for staining included anti-CD45, -CD3, -CD4, -CD8, -CD45RA, -CD197, -CD25, -CD127, -CD69, -41BB, -ICOS, -OX40, -LIGHT, -PD1, -TIGIT, -TIM3, -LAG3 (BioLegend). hCART were stained with cocktails of fluorescently-conjugated monoclonal antibodies (mAbs) or isotype-matched controls, cells were acquired on NovoCyte flow cytometer and analyzed using NovoExpress software (Agilent). Memory T cells were analyzed by gating for CD45RA/CD197 on CD45^+^/CD3^+^/CD4^+^ or CD45^+^/CD3^+^/CD8^+^ T cells. Co-stimulatory receptor expression was examined by gating for 41BB/ICOS/OX40/LIGHT on CD45^+^/CD3^+^/CD4^+^ or CD45^+^/CD3^+^/CD8^+^ T cells; and co-inhibitory receptor expression was examined by gating for PD-1/TIGIT/TIM3/LAG3 on CD45^+^/CD3^+^/CD4^+^ or CD45^+^/CD3^+^/CD8^+^ T cells.

### Statistical Analysis

All experiments were repeated at least 2-3 times and each condition had at least three replicates. The data are expressed as the means ± SD. Comparisons among groups were performed using ANOVA, and the comparisons within groups were performed using the Bonferroni and the Dunnett’s method. Multiple comparison were performed using Two-way ANOVA and Turkey’s multiple comparisons test. All statistical analyses were performed using GraphPad Prism version 8.0 software (GraphPad Software, Inc.). P<0.05 was considered as a statistically significant difference.

## Results

### Memory Phenotyping of hCART in Extended Cultures

After 1 month of culture, phenotyping of 3 sets of hCART generated from 2 donors showed (**Figures 1B–E**) very low CD4/CD8 ratios of 0.038 in COATC, 0.037 in hCART_ICOSζ_, and 0.062 in hCART_ICOS-27ζ_ compared to CD4/CD8 ratios of 0.38 for hCART_41BBζ_ and 0.87 for ATC (**Figure 1B**). Further analysis of CD4^+^ and CD8^+^ T cells in hCART and COATC showed a higher proportion of effector memory T cells that re-express CD45RA (T_EMRA_ cells) in CD8^+^ T cells in contrast to ATC that have a dominant population of T effector memory cells (T_EM_) (**Figure 1C**, right panel). The CD4^+^ T cells had equal proportions of T_EMRA_ and T_EM_ in all three hCART and COATC while the dominant population in ATC was T_EM_ (**Figure 1C**, left panel). Central memory (T_CM_) in CD4^+^ ranged between ~1-8% among hCART and COATC with 8% T_CM_ in ATC.

### CD8^+^ hCART Express High Levels of Co-Stimulatory and Low Levels of Co-Inhibitory Receptors After Long Term Expansion

Untransduced COATC, ATC and 41BBζ, ICOSζ, or ICOS-CD27ζ transduced hCART were stained for co-stimulatory and co-inhibitory receptors after long-term expansion with IL-2. High proportion of CD8^+^ hCART transduced with the above mentioned constructs showed high expression (~30-40%) of ICOS and OX40, ~2-10% expression of LIGHT and 1-8% expression of 41BB in CD8^+^ T cells. In the ATC population, the dominant co-stimulatory receptor expression was OX40 and low expression of LIGHT and 41BB (~1-5%) and no expression of ICOS was seen (**Figure 1D**, right panel). CD4^+^ hCART (41BBζ, ICOSζ, ICOS-CD27ζ) showed 2-15% staining for ICOS and 1-10% for OX40. ATC were positive for OX40 (~5%) and COATC showed <1% of ICOS and OX40 (**Figure 1D**, left panel).

The proportion of co-inhibitory receptors PD1, TIGIT, TIM3, LAG3 were less than 2% (ranged 0.5-1.5%) on CD4^+^ hCART populations and COATC, whereas ATC showed much higher proportion of co-inhibitory receptors (ranged 0.5-4.5%) on CD4^+^ ATC (**Figure 1E**, left panel). Similarly, the proportion of PD-1 and TIM3 co-inhibitory receptors on CD8^+^ T cells were <5% in hCART and COATC. CD8^+^ ATC showed 7.5% positivity for TIM3 expression (**Figure 1E**, right panel).

### Enhanced Specific Cytotoxicity by HER2 hCART Transduced With 41BBζ, ICOSζ and ICOS-27ζ Constructs

**Figure 2:**
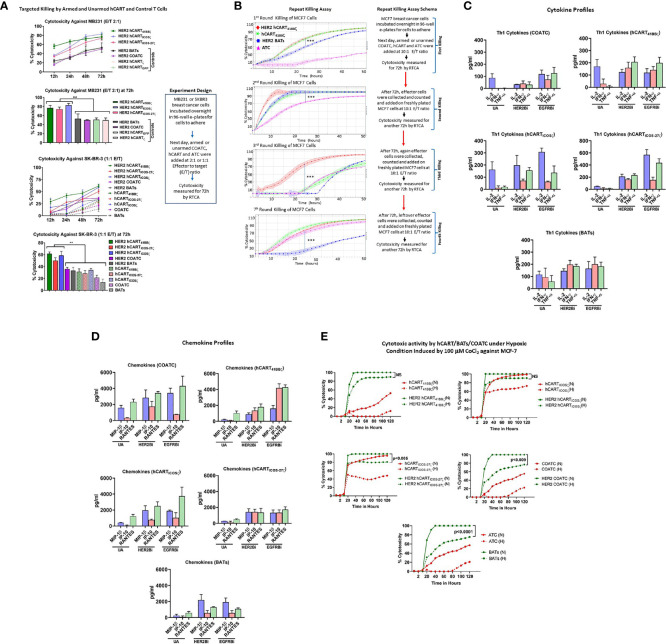
**(A)** Specific Cytotoxicity by hCART Transduced with Different Vectors. Upper panel showing specific cytotoxicity by Headless CAR T cells (hCART) transduced by different constructs against MB231 from 12 to 72 hours at E:T of 2:1 using the real time cell analysis (RTCA) by xCelligence System (n=4, each condition had 3-4 replicates). The statistical analysis at 72 hours show significantly high specific cytotoxicity by HER2 bispecific antibody armed hCART_41BBζ_ (HER2 hCART_41BBζ_; p<0.008), HER2 hCART_ICOSζ_ (p<0.003) and HER2 hCART_ICOS-27ζ_ (p<0.009) compared to controls-HER2 hCART_ζ_, HER2 hCART_GFP_, HER2 bispecific antibody armed co-activated T cells (COATC) and HER2 bispecific antibody armed T cells (BATs) against MB231 cells (n=3, each condition had 4 replicates). Data is presented as mean ± SD of 4 replicates overtime and analyzed using Two-way ANOVA and Turkey’s multiple comparisons test. Lower panel shows the kinetics of cytotoxicity against SKBR3 cells by HER2Bi armed hCART, COATC, BATs and unarmed hCART, COATC and ATC controls from 12-72 hours. At 72h, specific cytotoxicity was significantly high by HER2 hCART_41BBζ_ (p<0.001), HER2 hCART_ICOSζ_ (p<0.001), HER2 hCART_ICOS-27ζ_ (p<0.005) compared to HER2 COATC, HER2 BATs and unarmed hCART controls at E:T of 1:1 (n=4, each condition had 3 replicates). Data is presented as mean ± SD of 4 replicates overtime and analyzed using Two-way ANOVA and Turkey’s multiple comparisons test. **(B)** Serial Killing by hCART_41BBζ_ and BATs. Shows specific cytotoxicity (Mean ± SD) by HER2 bispecific antibody armed hCART_41BBζ_ (HER2 hCART_41BBζ_), HER2 BATs, unarmed (without BiAb loading) hCART_41BBζ_ (green) and ATC (pink) against MCF-7 cell line measured by RTCA up to 4 rounds of serial killing (n=3, each condition had 4-6 replicates). Each killing was monitored up to 50 hours at E:T of 10:1. HER2 hCART_41BBζ_ in all four killing rounds showed significantly higher cytotoxicity (p<0.0001) compared to one or more effector cells. Right panel shows the schema for repeat killing assay. Data is presented as mean ± SD of 4 replicates overtime and analyzed using Wilcoxon matched-pairs signed rank test. **(C)** Quantitative Cytokine Profiles of Culture Supernatants from Target (SKBR3) and Effector cells Co-culture. Th1 cytokines IL-2, IFN-γ and TNF-α in the culture supernatants are shown for unarmed (without BiAb loading) or armed with HER2Bi or EGFRBi hCART_41BBζ_, hCART_ICOSζ_, hCART_ICOS-CD27ζ_, COATC, ATC in the presence of target cells (SKBR3) co-cultures (n=2, each pooled from three replicates). Detailed statistical analysis is presented in **Table S1**. **(D)** Quantitative Chemokine Profiles of Culture Supernatants. Shows the chemokine in the culture supernatant from same co-cultures described above. Detailed statistical analysis is presented in **Table S1** **. (E)** Effect of Hypoxia on Effector Cell Functions. Showing cytotoxic activity by unarmed and HER2Bi armed hCART, COATC, ATC under normoxic (N) and hypoxic (H) conditions induced by 100 µM CoCl_2_ against MCF-7 cell line (n=3, each condition had 4-6 replicates). There were no significant functional difference found between normoxic (solid lines) and hypoxic (dashed lines) conditions on the cytotoxic effects of HER2 hCART_41BBζ_ and HER2 hCART_ICOSζ_ against MCF-7 cells in long term (120 hours) killing assay. Cytotoxicity by BATs (p<0.0001), COATC (p<0.009) and headless CAR T cells with ICOS-27ζ intracellular domain was significantly lower (p<0.005) under hypoxic condition compared to normoxia against MCF-7 cells. Data is presented as mean of 6 replicates overtime and analyzed using Wilcoxon matched-pairs signed rank test (***p < 0.0001, **p < 0.009.).

Specific cytotoxicity mediated by HER2 hCART_41BBζ_, hCART_ICOSζ_ or hCART_ICOS-27ζ_ compared to hCART transduced with GFP (hCART_GFP_) or ζ (hCART_ζ_), untransduced HER2 COATC and HER2 BATs was measured by using RTCA against low HER2 expressing MB231 cancer cells. Specific cytotoxicity by HER2 hCART_41BBζ_, HER2 hCART_ICOSζ,_ or HER2 hCART_ICOS-27ζ_ ranged between 60-80% compared to HER2 hCART_GFP_, HER2 hCART_ζ,_ HER2 COATC and HER2 BATs that showed ~40-45% cytotoxicity at an E:T of 2:1 against MB231 cells at 72 hours (**Figure 2A**, upper panel). Multiple comparision by one way ANOVA comparing mean cytotoxicity of HER2 hCART_41BBζ_, HER2 hCART_ICOSζ,_ or HER2 hCART_ICOS-27ζ_ with means of HER2 hCART_GFP_, HER2hCART_ζ_, HER2 COATCs and HER2 ATC showed significantly increased cytotoxicity by HER2 hCART_41BBζ_ (p<0.008), HER2 hCART_ICOSζ,_ (p<0.003) and HER2 hCART_ICOS-27ζ_ (p<0.009) at 72 hours (**Figure 2A**, lower bar graph). Lower panel of **Figure 2A** shows the kinetics of cytotoxicity by HER2 hCART_41BBζ_, HER2 hCART_ICOSζ_ or HER2 hCART_ICOS-27ζ_ compared to HER2 COATC, HER2 BATs and unarmed hCART controls from 12-72 hours. The statistical analysis at 72h show significantly increased specific cytotoxicity by HER2 hCART_41BBζ_ (p<0.001), HER2 hCART_ICOSζ_ (p<0.001), or HER2 hCART_ICOS-27ζ_ (p<0.005) ranged between 50-60% compared to HER2 COATC, HER2 BATs and unarmed hCART controls that ranged from ~12-35% against SKBR3 cells at an E:T of 1:1. Right panel shows the experimental design. These results were reproducible using effectors derived from 2 normal donors targeting multiple cell lines.

### Comparison of Serial (Repeated) Killing by Unarmed or BiAb Armed hCART, COATC and ATC

Serial cytotoxicity (Mean ± SD; n=3) against MCF-7 cells by HER2 hCART_41BBζ, _unarmed hCART_41BBζ, _(hCART_41BBζ_), HER2 BATs and unarmed ATC (ATC) monitored by RTCA are shown in **Figure 2B**. After each killing round, effector cells were re-plated on new targets up to 4^th^ killing assay with repeated transfers onto fresh MCF-7 cells on days 3, 7, 10 and 14 at an E:T of 10:1 as described (23). In the 1^st^ round of killing, at 24 hours (h) both HER2 hCART_41BBζ _and hCART_41BBζ _showed significantly higher killing (P<0.0001) than HER2 BATs. By 45-50h, 90-100% cytotoxicity was reached by HER2 hCART_41BBζ _, hCART_41BBζ_ and HER2 BATs while ATC showed ~10% cytotoxicity. In a 2^nd^ round of killing, HER2 hCART_41BBζ _, hCART_41BBζ_ and HER2 BATs reached 100% cytotoxicity within 24h, cytotoxicity was significantly higher killing (P<0.0001) compared to ATC that showed >70% at 24h. By 3^rd^ round, cytotoxicity was less than 10% at 24h by hCART_41BBζ_ HER2 BATs and ATC compared to significantly high killing (P<0.0001) by HER2 hCART_41BBζ _(~70%), however at 50h, mean cytotoxicity reached 70-88% by hCART_41BBζ_, HER2 BATs and ATC. Interestingly, by 4^th^ round of serial killing, cytotoxicity hCART_41BBζ _and ATC is equal to HER2 hCART_41BBζ _and surpass HER2 BATs (**Figure 2B**). Right panel of **Figure 2B** shows the schema for repeat killing assay. These data are consistent with previously reported study (23) that there may be a selective expansion of memory cells and confirm the notion that unarmed hCART and ATC were repeatedly primed *in vitro* with antigens released during repeated killing of tumor cells. Since the BiAb loaded on the surface of the effectors is diluted by cell divisions, therefore, tumor engagement capacity of the BiAb is concomitantly diluted and should result in an expected decrease in specific cytotoxicity. The unexpected increased cytotoxicity at fourth round of killing by unarmed effector cells may be due to expansion of memory clones as a result of repeated antigenic exposure (*in vitro immunization*) or to the emergence of innate recognition molecules such as NKG2C capable of T cell receptor (TCR)-dependent and TCR-independent release of cytotoxic granule proteins. Serial killing by hCART directed at SKBR3 cell line showed similar repeat killing pattern (data not shown).

Mean cytotoxicity mediated by other unarmed effectors (hCART_ICOSζ_, hCART_ICOS-27ζ_, COATC, and ATC) and armed effectors (HER2 hCART_ICOSζ_, HER2 hCART_ICOS-27ζ_, HER2 COATC and HER2 BATs) was also measured by RTCA in a long-term serial killing assay directed at MCF-7, data is presented in **Figure S1**. Specific cytotoxicity reached 80-100% on the first killing cycle within 24 hours at an E:T of 10:1 against MCF-7 (**Figure S1**, upper left panel) and 100% within 10 hours in second killing cycle for all armed effector cells (**Figure S1**, upper right panel). By the third round, cytotoxicity reached ~70 - 100% at 72 hours for all armed effector cells but unarmed effector cells showed delayed cytotoxicity (40-80%) at 72 hours (**Figure S1**, lower left panel). Intriguingly, by the fourth round of killing, specific cytotoxicity was ~80% for both unarmed and HER2 hCART_41BBζ_, HER2 hCART_ICOSζ_, COATC and ATC by 72 hours (**Figure S1**, lower right panel). While armed ATC (HER2 BATs) showed transient killing up to 30 hours and declined thereafter.

### Engagement of HER2 hCART or EGFR hCART With Tumor Induces Cytokines and Chemokines

HER2Bi or EGFRBi armed hCART_41BBζ_, hCART_ICOS-27ζ_ and BATs induced robust levels of Th_1_ cytokines-IFN-γ and TNF-α upon tumor engagement at 1:1 E:T ratio compared to unarmed counterparts except for IL-2 that did not show any change in levels between armed hCART_41BBζ_ and BATs compared to unarmed hCART_41BBζ_ and BATs. Background levels produced by effectors without targets were subtracted from the data shown in **Figure 2C**.

Likewise, high levels of T cell recruiting and activating IFN-γ induced IP-10/CXCL10 chemokine was induced by EGFR hCART_41BBζ,_ during overnight co-culture with tumor cells at 1:1 E:T ratio compared other hCART, COATC and BATs. The levels of other T cell recruiting chemokines, MIP-1β and RANTES were higher in the supernatants from HER2- or EGFR hCART_41BBζ,_ hCART_ICOSζ_ and hCART_ICOS-27ζ_, COATC and BATs co-cultured with tumor targets at 1:1 E:T ratio compared to their corresponding unarmed hCART, COATC and ATC (**Figure 2D**).

### hCART_41BBζ_ and hCART_ICOSζ_ Are Resistant to *In Vitro* Hypoxic Condition

To determine the effect of hypoxia on the cytotoxicity activity of hCART_41BBζ_, hCART_ICOSζ_ and hCART_ICOS-27ζ_, hypoxia-mimetic cobalt chloride (CoCl_2_) was pretitrated on MCF-7 breast cancer cell line and COATC at doses from 50 µM to 400 µM (**Figure S1**). Hypoxia induced death of MCF-7 cells (**Figure S1**, upper panel) was measured by RTCA and hypoxia induced cytotoxicity of COATC was measured by flow cytometry using Annexin-V and 7-AAD staining (**Figure S1**, lower panel). Cell death at 100 µM CoCl_2_ was <10% for MCF-7 targets at 24 hours and cell death for COATC at 100 µM CoCl_2_ was <10% after 24 hours. Therefore, a dose of 100 µM dose of CoCl_2_ was selected for inducing hypoxia to determine whether hCART are functionally active under hypoxic stress. Interestingly, HER2 hCART_41BBζ_ and HER2 hCART_ICOSζ_ showed no significant functional difference between normoxia and hypoxia in long term (120 hours) cytotoxicity assays, cytotoxicity ranged between 80-100% against MCF-7 cells. However, cytotoxicity was significantly lower under hypoxic conditions for HER2 hCART_ICOS-27_ (p<0.005), HER2 COATC (p<0.009) and HER2 BATs (p<0.0001) against MCF-7 cells compared to normoxia. These data suggest that 41BBζ and ICOSζ intracellular domains are significantly better at resisting *in vitro* hypoxic stress than COATC and BATs. Representative data is shown in **Figure 2E**.

### Focus on Functional Characterization of hCART_41BBζ_

Based on phenotyping of T cell receptors and co-receptors especially in CD4^+^ T cells (T_EMTA_); functional screening for the optimal ICD for cytotoxic effector function; the resistance to hypoxia induced cell death; ability to function under hypoxic conditions and provide an effective *in vitro* immunization effect, we chose hCART_41BBζ_ for the next set of experiments.

### High Expression Levels of Transgene in hCART_41BBζ_

**Figure 3:**
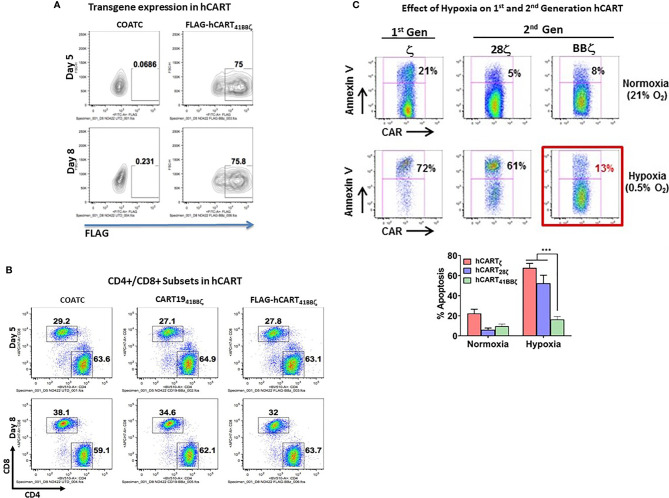
**(A)** Transduction Efficiency of COACT. Representative data from COATC with 41BBζ intracellular domain **(**hCART_41BBζ_) show 75% transgene expression (detected by anti-FLAG antibody) on day five and day eight. **(B)** CD4 and CD8 T cell Subsets in hCART. The CD4/CD8 T cells ratio in hCART_41BBζ_, CD19_41BBζ_ CAR-T cells and untransduced T cells (COATC) on day five and day eight suggest no change in CD4/CD8 T cells ratio due to transgene insertion including transduction of COATC with different intracellular signaling domains. **(C)** hCART_41BBζ are_ Resistant to *In Vitro* Hypoxia Effectors. Showing that hypoxia differentially affected survival of hCART depending on the co-stimulatory endodomain. Headless CAR T cells with ζ alone (hCART_ζ_) or CD28ζ endodomain (hCART_28ζ_) showed 72% and 61% apoptosis under hypoxia, respectively, whereas hCART_41BBζ_ showed just 13% apoptosis under hypoxic (0.5% oxygen) condition. Lower panel shows significantly less (p<0.0003) apoptosis of hCART_41BBζ _compared to hCART_ζ _or hCART_28ζ_ under hypoxic condition. Data is presented as mean ± SD of 3 experiments and analyzed using Two-way ANOVA and Turkey’s multiple comparisons test (***p < 0.0003).

Flow cytometry confirmed that T cells transduced with 41BBζ construct show high expression of 41BBζ in ~75% of CD4^+^/CD8^+^ T cells on days five and eight by detecting FLAG tag (**Figure 3A**). Proportion of CD4^+^ and CD8^+^ T cell populations of 41BBζ transduced T cells (hCART_41BBζ_) were comparable to the untransduced COATC and CD19-41BBζ CAR-T cells (CD19-BBζ). On day five, CD4^+^ and CD8^+^ T cells were 63.1% and 27.8% positive for FLAG positive hCART_41BBζ_, respectively; on day eight, 63.7% CD4^+^ and 32% CD8^+^ T cells were FLAG positive , respectively (**Figure 3B**).

### Resistance to Hypoxia and Exhaustion Is a Function of the 41BBζ Co-Stimulatory Endodomain of hCART

We next determined whether the hCART_41BBζ_ can survive in stringent hypoxic conditions using hCART_ζ_ or BTC_28ζ_ as controls. hCART_ζ_ or BTC_28ζ_ were 72% and 61% apoptotic under 0.5% oxygen, respectively, whereas only 13% of the hCART_41BBζ_ became apoptotic under hypoxic conditions (**Figure 3C**). These data suggest that survival in hypoxia was differentially affected by the co-stimulatory 28ζ or 41BBζ intracellular domains. Upper panel of **Figure 3C** shows the representative data from one experiment, bar graph below shows the composite data for sensitivity of hCART containing different constructs for hypoxia induced apoptosis (n=3). Data show significantly less (p<0.0003) apoptosis of hCART_41BBζ _compared to hCART_ζ _or hCART_28ζ_ under hypoxic condition.

The differential effects of hypoxia on hCART with 28ζ and 41BBζ are reflective of differential metabolic activity of hCART transduced with 28ζ or 41BBζ. The metabolic reprogramming of hCART expressing either 28ζ or 41BBζ intracellular domains on day 0 or day 7 are shown in **Figure S3**, demonstrating enhanced basal oxygen consumption rates (OCR) with augmented spare respiratory capacities of hCART_41BBζ_ than hCART_28ζ_ on day 7 of culture. The metabolic features of hCART were measured by a sea-horse assay.

### HER2 hCART_41BBζ_ Targets High and Low HER2 or EGFR Expressing Cell Lines

**Figure 4:**
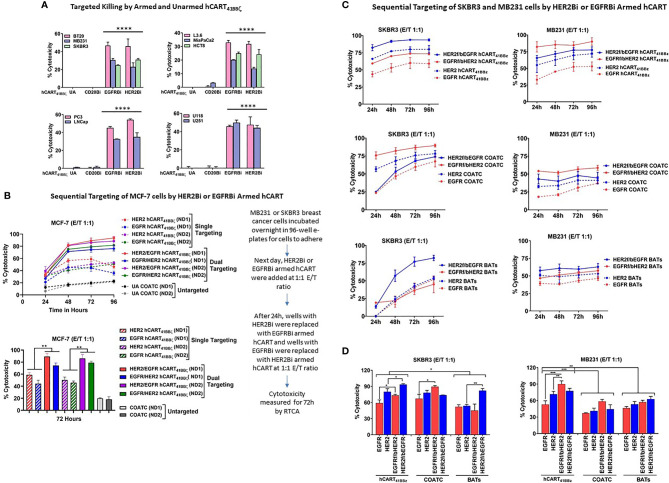
hCART_41BBζ_: **(A)** Specific Cytotoxicity by HER2 hCART_41BBζ_ and EGFR hCART_41BBζ_ Directed at Solid Tumor Cell Lines. The four panels show cytotoxicity of unarmed COATC (Control), CD20 hCART_41BBζ_ (irrelevant control), EGFR hCART_41BBζ_ and HER2 hCART_41BBζ_ in ^51^Cr release assay at E:T of 10:1. Cytotoxicity was measured against breast (Upper left), pancreas/GI (Upper right), prostate (Lower left) and glioblastoma (Lower right) cell lines. Data is presented as mean ± SD (n=3) and analyzed using Two-way ANOVA and Turkey’s multiple comparisons test (****p < 0.00001). **(B)** Sequential Targeting of MCF-7 with HER2 hCART_41BBζ_ and EGFR hCART_41BBζ_. Upper panel shows the sequential cytotoxicity against MCF-7 cell line with either HER2 hCART_41BBζ_ followed by (f/b) EGFR hCART_41BBζ_ or EGFR_41BBζ_ (f/b) HER2 hCART_41BBζ_ using RTCA up to 96 hours at E:T of 1:1. The dashed lines denote single antigen targeting by HER2 hCART_41BBζ_ or EGFR hCART_41BBζ_ and solid lines show the double sequential killing mediated by effectors from 2 normal donors (ND1 =Normal Donor 1 and ND2 = Normal Donor 2). Lower panel shows the significant killing at 72 hours with sequential antigen targeting by HER2 hCART_41BBζ_ f/b EGFR hCART_41BBζ_ or EGFR hCART_41BBζ_ f/b HER2 hCART_41BBζ_ of MCF-7 compared to single antigen targeting by HER2 hCART_41BBζ_ (p<0.007) and EGFR hCART_41BBζ_ (p<0.003). Data is presented as mean ± SD (n=3) and analyzed using Two-way ANOVA and Turkey’s multiple comparisons test (**p < 0.001*). Right panel shows the sequential targeting schema. **(C)** Show Sequential Targeting of Two Antigens in SKBR3 and MB231 Cell Lines. The hCART_41BBζ_ induced significantly higher cytotoxicity against SKBR3 cells by sequential targeting of HER2 f/b EGFR (p<0.03) or EGFR f/b HER2 (p<0.02) compared to single antigen targeting. COATC and BATs also showed significantly enhanced killing by sequential targeting of EGFR f/b HER2 (p<0.03), HER2 f/b EGFR (p<0.004) compared single antigen targeting on SKBR3 cell line (Left panels of C and D). Significantly higher cytotoxicity after sequential targeting of EGFR f/b HER2 by hCART_41BBζ_ compared to single antigen targeting of HER2 (p<0.001) or EGFR (p<0.0002) was observed for MB231 cells (Right panels of **C, D**). Data is presented as mean ± SD (n=3) and analyzed using Two-way ANOVA and Turkey’s multiple comparisons test (***p < 0.0001, **p < 0.001, *p < 0.01).

To determine the specific cytotoxicity of HER2 hCART_41BBζ_ and EGFR hCART_41BBζ _ against HER2 and EGFR expressing breast cancer (low HER2 expressing BT20 and MB231 and high HER2 expressing SKBR3 cell lines), pancreatic cancer, prostate cancer and glioblastoma cell lines, the effectors were tested in ^51^Cr release assays at E:T of 10:1 (**Figure 4A**). HER2 hCART_41BBζ_ and EGFR hCART_41BBζ _exhibited significantly higher cytotoxicity directed at BT20, MB231, and SKBR3 (p<0.00001) breast cancer lines (**Figure 4A**, left upper panel), L3.6, MiaPaCa2 and HCT8 (p<0.00002) pancreatic cancer lines (**Figure 4A**, right upper panel), cytotoxicity (35-55%) directed at PC3, and LNCap (p<0.00001) prostate cancer lines (**Figure 4A**, lower left panel), and roughly 40% cytotoxicity directed at U118, and U251 (p<0.00001) glioblastoma cell lines (**Figure 4A**, lower right panel) compared unarmed hCART_41BBζ _(UA) and hCART_41BBζ_ armed with irrelevant anti-CD3 x anti-CD20 BiAb (CD20Bi). It is noteworthy that HER2 hCART_41BBζ_, as previously reported for HER2 BATs, exhibited cytotoxicity against low HER2 expressing BT20 and MB231 cell lines comparable to cytotoxicity against the high HER2 expressing SKBR3 cell line; these findings confirm that only a few molecules of antigens on target cells are sufficient to bind and trigger cytotoxicity (15).

### Enhanced Cytotoxicity by HER2 hCART_41BBζ _and EGFR hCART_41BBζ _in Double Sequential Targeting of MCF-7 Targets

We posited that sequential killing by HER2 hCART_41BBζ_ followed by (f/b) EGFR hCART_41BBζ_ or EGFR hCART_41BBζ_ f/b HER2 hCART_41BBζ_ would decrease antigen escape and enhance cytotoxicity above that is mediated by EGFR hCART_41BBζ_ or HER2 hCART_41BBζ_ alone. We selected MCF-7 cells as targets since these cells express low levels of HER2 and EGFR to provide proof of concept for targeting of tumors that express low levels of antigen (**Figure 4B**, upper panel). The sequential addition of HER2 hCART_41BBζ_ f/b EGFR hCART_41BBζ_ or EGFR hCART_41BBζ_ f/b HER2 hCART_41BBζ_ exhibited significantly higher (p<0.007 and p<0.003) specific cytotoxicity in two normal donors (ND1 and ND2) compared to killing mediated by single antigen targeting at a low E:T of 1:1 (n=2) in the presence of 100 IU/mL IL-2 at 72 hours (**Figure 4B**, lower panel).

Similar to MCF-7, significantly higher cytotoxicity was found after sequential targeting of two antigens by HER2 hCART_41BBζ_ f/b EGFR hCART _41BBζ_ (p<0.03) or EGFR f/b HER2, p<0.02) compared to single antigen targeting on SKBR3 cell line. COATC also showed significantly enhanced killing in the sequential targeting of EGFR f/b HER2 compared to single targeting of EGFR antigen (p<0.03); similarly, targeting with HER2 BATs f/b EGFR showed significantly enhanced killing (p<0.004) compared to single targeting of HER2 expressed on SKBR3 cell line (**Figures 4C, D**, left and lower panels). Comparable patterns of significantly higher cytotoxicity were seen in MB231 cells after sequential targeting of EGFR hCART_41BBζ_ f/b HER2 hCART_41BBζ_ compared to single antigen targeting of HER2 (p<0.001) or EGFR (p<0.0002). Interestingly, hCART_41BBζ_ mediated sequential or single targeting cytotoxicity was significantly higher compared to COATC (p<0.0005) or BATs (p<0.004) against SKBR3 and MB231 **(**BATs, p<0.05) (**Figures 4C, D**, right and lower panels).

## Discussion

The solid TME is a formidable challenge that includes the physical barrier of cancer stroma, an altered metabolic landscape, nutrient insufficiency, regulatory cells and cytokines and chemokines derived from tumors that inhibit adoptively transferred T cells to proliferate, persist and function in the TME (24–26). Metabolic profiling of T cells from tumors reveals markedly depressed oxidative function and upregulation of co-inhibitory molecules such as PD-1, TIM-3, and LAG-3 which correlate with both T cell dysfunction and metabolic insufficiency (27–29). We designed the constructs to enhance metabolic activity of *ex vivo* expanded hCART.

HER2 hCART_41BBζ_ or EGFR hCART_41BBζ_ exhibit high levels of specific cytotoxicity directed at multiple tumor targets that express high and low antigen levels. It is reassuring to confirm that sequential targeting of two distinct antigens led to significantly higher cytotoxicity than targeting a single antigen. These data support the strategy of dual targeting to avoid the clonal escape. In long-term cultures involving repetitive exposure to tumor, unarmed hCART, COATC and ATC developed specific cytotoxicity that was equal to or comparable to that exhibited by HER2 hCART_41BBζ, _HER2 hCART_ICOSζ_, and HER2 BATs after three rounds of exposure to antigen and repeat killing. These results show that repeated exposure to tumor antigens *in vitro* can lead to primary immunization and expansion of T effector memory clones against tumor associated antigens (TAA) in the culture (30).

The BiAb armed non-MHC restricted killing in hypoxic condition mediated by metabolically enhanced hCART provides a novel and highly adaptable platform for serial or sequential targeting of multiple antigens on tumors and the ability to select different BiAbs targeting multiple TAAs. These features provide the platform for the next generation of more potent targeted effector T cells for immunotherapy in solid tumors.

While co-stimulatory signaling endodomains play key roles in persistence and effector functions of CAR-T cells (31–33), the composition and length of the non-signaling, non-targeting flexible hinge can influence the strength of the CAR response (34). CAR-T cells with CD28ζ domain show rapid effector functions but decreased persistence while CAR-T_41BBζ_ cells show sustained effector functions with increased persistence and enriched T_CM_ (32). Similar to CAR-T_41BBζ_ cells, hCART_41BBζ_ show greater resistance to hypoxia induced apoptosis compared to hCART_28ζ_ (13% *vs*. 61% apoptosis under 0.5% O_2_). The TME is often hypoxic, O_2_ ranges 0.3% to 2.1% O_2_ depending on tumor type (35–37) compared to the normoxic condition of 20% O_2_ in *in vitro* 2D cultures (38). This study shows that the cytotoxicity mediated by 2 out 3 armed hCART (HER2 hCART_41BBζ_ and HER2 hCART_ICOSζ_) under hypoxia had no effect on their cytotoxicity compared to normoxic condition. However, HER2 hCART_ICOS-27ζ_, COATC and ATC all had significantly lower cytotoxicity under hypoxic conditions compared to normoxic conditions. These data suggest that hCART transduced with 41BBζ and ICOSζ ICD are able to survive and kill tumor cells *in vitro* under hypoxic condition.

It is not surprising to see induction of Th_1_ (IL-2 and IFN-γ) cytokines by unarmed (UA) activated T cells (ATC) co-cultured with tumor cells. The fact that UA ATC exhibit non-MHC restricted cytotoxicity against tumor cells indicate that variable levels of IL-2 and IFN-γ are likely to be produced for their proliferation and anti-tumor activity. Although the cytotoxicity by UA ATC against tumor cells is lower compared to armed T cells, but may induce comparable levels of IL-2 (IL-2 in the culture supernatants of UA ATC and tumor cells ranges: 47-170 pg/ml). Whereas levels of IFN-γ are usually low (IFN-γ in the culture supernatants of UA ATC and tumor cells ranges: 0-92 pg/ml) compared to armed T cells, which is consistent with our previous studies showing induction of IFN-γ in UA ATC + tumor cells co-cultures (39, 40). Likewise studies have shown that unarmed ATC exhibit cytotoxicity directed at hematologic and solid tumors (41–43). We translated this product in the clinic by infusing UA ATC after stem cell transplant (SCT) in women with metastatic breast cancer (44) and UA ATC infusions after immunization with BATs can transfer antigen specific killer cell activity into patients after myeloablative therapy and autologous SCT (16).

The activation mechanism of “headless CAR” construct is likely through TCR signaling that may transcostimulate intracellular “headless CAR” construct either through intracellular signaling molecules or through external stimuli such as binding of IFN-γ to its receptor (positive feedback mechanism), inducing intracellular signaling such as JAK/STAT pathway. Other studies have also shown this transcostimulation phenomenon delivered by small molecule-mediated aggregation of the transmembrane and cytoplasmic MyD88-CD40 domains, which, imparts critical functionality to the various CAR constructs (45). The likelihood of transcostimulation with engagement of the T cells bearing a myriad of co-stimulatory receptors and “forced” engagement by highly crosslinked TCR with the BiAb binding not only with target antigen but also number of ligands on tumor cells to co-stimulatory receptors on T cells providing additional transcostimulation to the “CAR-less” construct.

We deleted the CAR of the construct to minimize the possibility of cytokine release syndrome by eliminating tonic stimulation by tumor antigen and establish an adaptable and flexible targeting platform. The metabolically enhanced hCART could be armed with off-the-shelf BiAb(s) to target one or more antigens on solid tumors sequentially or simultaneously. This exogenous targeting approach avoids the necessity for engineering a different or multiple CAR(s) for each tumor type. This approach has an “autobrake” built-in to the effector cells since the concentration of BiAb loaded onto the surface hCART is fixed and dilutes out with each cell division; after multiple rounds of repeated killing (typically 2-3 weeks),thus avoiding tonic cytokine/chemokine stimulation leading to CRS. This self-limiting governance of antigen-engagement would not only limit toxicity but will also allow for multiple infusions. An advantage of the BiAb loading of hCART is that commercial antibodies (Herceptin^®^, Erbitux^®^, etc) can be quickly derivatized into BiAbs with OKT3 or existing recombinant BiAbs can be loaded. Thus, BiAb armed hCART offer enhanced survivability and anti-tumor cytotoxicity with metabolic fitness and flexibility for targeting one or more tumor antigens in the TME with built-in CRS regulator.

In a phase I trial in 23 metastatic breast cancer patients and 7 hormone refractory prostate cancer patients (15, 17, 18), HER2 BATs were safe without dose limiting toxicities in the outpatient setting, and induced a partial remission in a HER2 negative patient for 7.5 months (15). For the entire group, HER2 negative group, and HER2 positive group of breast cancer patients, the median overall survival was 36, 27, and 57 months, respectively. In a phase I/II in a series of seven patients with unresectable or metastatic pancreatic cancer, EGFR BATs infusions induced clinical responses with stable disease for 7.4 months, two complete remissions to chemotherapy given after EGFR BATs, and one complete response survivor at 60 months (18). These encouraging clinical signals in metastatic breast and pancreatic cancer provide a strong rationale for engineering armed hCART for a phase I trial for solid tumors.

In summary, our preclinical studies show that HER2 or EGFR hCART_41BBζ_ can effectively kill multiple tumor targets, kill tumor targets in sequential and serial killing assays, secrete Th_1_ cytokines and chemokines and exhibit specific target killing in *in vitro* hypoxic condition.

## Data Availability Statement

The original contributions presented in the study are included in the article/**Supplementary Material**. Further inquiries can be directed to the corresponding authors.

## Author Contributions 

LL, CJ, and AT conceived the idea. AT and LL share lead authorship. AT, LL, CJ, and JS designed the study and performed the statistical analysis. AT, JS, EK, EB, and DS performed the experiments and participated in the data analysis. All authors contributed to the article and approved the submitted version.

## Funding

This study was primarily supported by funding from in part by R01-CA92344, R01-CA140314, R0-CA182526, and P30-CA022453 (Microscopy, Imaging, and Cytometry Resources Core) and startup funds from the University of Virginia Cancer Center, R01-CA226983 (CJ) from the University of Pennsylvania.

## Conflict of Interest

LL is co-founder of *Transtarget* Inc. and serves on the SAB for Rapa Therapeutics, CJ is a co-founder of *Tmunity Therapeutics*, Inc. and AT is co-founder of *AlphaImmunePlatform LLC*.

The remaining authors declare that the research was conducted in the absence of any commercial or financial relationships that could be construed as a potential conflict of interest.

## References

[1] Brentjens RJ Riviere I Park JH Davila ML Wang X Stefanski J. Safety and Persistence of Adoptively Transferred Autologous CD19-Targeted T Cells in Patients With Relapsed or Chemotherapy Refractory B-Cell Leukemias. Blood (2011) 118(18):4817–28. 10.1182/blood-2011-04-348540 PMC3208293 21849486

[2] Rosenberg SA Restifo NP. Adoptive Cell Transfer as Personalized Immunotherapy for Human Cancer. Science (2015) 348(6230):62–8. 10.1126/science.aaa4967 PMC6295668 25838374

[3] Porter DL Levine BL Kalos M Bagg A June CH. Chimeric Antigen Receptor-Modified T Cells in Chronic Lymphoid Leukemia. N Engl J Med (2011) 365(8):725–33. 10.1056/NEJMoa1103849 PMC3387277 21830940

[4] Guedan S Ruella M June CH. Emerging Cellular Therapies for Cancer. Annu Rev Immunol (2019) 37:145–71. 10.1146/annurev-immunol-042718-041407 PMC7399614 30526160

[5] Brown CE Alizadeh D Starr R Weng L Wagner JR Naranjo A. Regression of Glioblastoma After Chimeric Antigen Receptor T-Cell Therapy. N Engl J Med (2016) 375(26):2561–9. 10.1056/NEJMoa1610497 PMC5390684 28029927

[6] Ahmed N Brawley V Hegde M Bielamowicz K Kalra M Landi D. HER2-Specific Chimeric Antigen Receptor-Modified Virus-Specific T Cells for Progressive Glioblastoma: A Phase 1 Dose-Escalation Trial. JAMA Oncol (2017) 3(8):1094–101. 10.1001/jamaoncol.2017.0184 PMC5747970 28426845

[7] Kosti P Maher J Arnold JN. Perspectives on Chimeric Antigen Receptor T-Cell Immunotherapy for Solid Tumors. Front Immunol (2018) 9:1104. 10.3389/fimmu.2018.01104 29872437 PMC5972325

[8] Martinez M Moon EK. CAR T Cells for Solid Tumors: New Strategies for Finding, Infiltrating, and Surviving in the Tumor Microenvironment. Front Immunol (2019) 10:128. 10.3389/fimmu.2019.00128 30804938 PMC6370640

[9] Geiger R Rieckmann JC Wolf T Basso C Feng Y Fuhrer T. L-Arginine Modulates T Cell Metabolism and Enhances Survival and Anti-Tumor Activity. Cell (2016) 167(3):829–42.e13. 10.1016/j.cell.2016.09.031 27745970 PMC5075284

[10] Chang CH Qiu J O’Sullivan D Buck MD Noguchi T Curtis JD. Metabolic Competition in the Tumor Microenvironment Is a Driver of Cancer Progression. Cell (2015) 162(6):1229–41. 10.1016/j.cell.2015.08.016 PMC4864363 26321679

[11] Ben-Shoshan J Maysel-Auslender S Mor A Keren G George J. Hypoxia Controls CD4+CD25+ Regulatory T-Cell Homeostasis *via* Hypoxia-Inducible Factor-1alpha. Eur J Immunol (2008) 38(9):2412–8. 10.1002/eji.200838318 18792019

[12] Patsoukis N Bardhan K Chatterjee P Sari D Liu B Bell LN. PD-1 Alters T-Cell Metabolic Reprogramming by Inhibiting Glycolysis and Promoting Lipolysis and Fatty Acid Oxidation. Nat Commun (2015) 6:6692. 10.1038/ncomms7692 25809635 PMC4389235

[13] Di Stasi A Tey SK Dotti G Fujita Y Kennedy-Nasser A Martinez C. Inducible Apoptosis as a Safety Switch for Adoptive Cell Therapy. N Engl J Med (2011) 365(18):1673–83. 10.1056/NEJMoa1106152 PMC3236370 22047558

[14] Lamers CH Sleijfer S van Steenbergen S van Elzakker P van Krimpen B Groot C. Treatment of Metastatic Renal Cell Carcinoma With CAIX CAR-Engineered T Cells: Clinical Evaluation and Management of On-Target Toxicity. Mol Ther (2013) 21(4):904–12. 10.1038/mt.2013.17 PMC5189272 23423337

[15] Lum LG Thakur A Al-Kadhimi Z Colvin GA Cummings FJ Legare RD. Targeted T-Cell Therapy in Stage IV Breast Cancer: A Phase I Clinical Trial. Clin Cancer Res (2015) 21(10):2305–14. 10.1158/1078-0432.CCR-14-2280 PMC4433762 25688159

[16] Thakur A Rathore R Kondadasula SV Uberti JP Ratanatharathorn V Lum LG. Immune T Cells Can Transfer and Boost Anti-Breast Cancer Immunity. Oncoimmunology (2018) 7(12):e1500672. 10.1080/2162402X.2018.1500672 30524893 PMC6279339

[17] Vaishampayan U Thakur A Rathore R Kouttab N Lum LG. Phase I Study of Anti-CD3 X Anti-Her2 Bispecific Antibody in Metastatic Castrate Resistant Prostate Cancer Patients. Prostate Cancer (2015) 2015:285193. 10.1155/2015/285193 25802762 PMC4352947

[18] Lum LG Thakur A Choi M Deol A Kondadasula V Schalk D. Clinical and Immune Responses to Anti-CD3 X Anti-EGFR Bispecific Antibody Armed Activated T Cells (EGFR BATs) in Pancreatic Cancer Patients. Oncoimmunology (2020) 9(1):1773201. 10.1080/2162402X.2020.1773201 32939319 PMC7480816

[19] Thakur A Schalk D Sarkar SH Al-Khadimi Z Sarkar FH Lum LG. A Th1 Cytokine-Enriched Microenvironment Enhances Tumor Killing by Activated T Cells Armed With Bispecific Antibodies and Inhibits the Development of Myeloid-Derived Suppressor Cells. Cancer Immunol Immunother (2012) 61(4):497–509. 10.1007/s00262-011-1116-1 21971587 PMC3800101

[20] Thakur A Schalk D Tomaszewski E Kondadasula SV Yano H Sarkar FH. Microenvironment Generated During EGFR Targeted Killing of Pancreatic Tumor Cells by ATC Inhibits Myeloid-Derived Suppressor Cells Through COX2 and PGE2 Dependent Pathway. J Transl Med (2013) 11:35. 10.1186/1479-5876-11-35 23394575 PMC3608954

[21] Sen M Wankowski DM Garlie NK Siebenlist RE Van Epps D LeFever AV. Use of Anti-CD3 X Anti-HER2/neu Bispecific Antibody for Redirecting Cytotoxicity of Activated T Cells Toward HER2/neu+ Tumors. J Hematother Stem Cell Res (2001) 10(2):247–60. 10.1089/15258160151134944 11359672

[22] Grabert RC Cousens LP Smith JA Olson S Gall J Young WB. Human T Cells Armed With Her2/neu Bispecific Antibodies Divide, Are Cytotoxic, and Secrete Cytokines With Repeated Stimulation. Clin Cancer Res (2006) 12(2):569–76. 10.1158/1078-0432.CCR-05-2005 16428502

[23] Thakur A Scholler J Schalk DL June CH Lum LG. Enhanced Cytotoxicity Against Solid Tumors by Bispecific Antibody-Armed CD19 CAR T Cells: A Proof-of-Concept Study. J Cancer Res Clin Oncol (2020) 146(8):2007–16. 10.1007/s00432-020-03260-4 PMC7375514 32449004

[24] Delgoffe GM Powell JD. Feeding an Army: The Metabolism of T Cells in Activation, Anergy, and Exhaustion. Mol Immunol (2015) 68(2 Pt C):492–6. 10.1016/j.molimm.2015.07.026 PMC4837657 26256793

[25] Scharping NE Menk AV Moreci RS Whetstone RD Dadey RE Watkins SC. The Tumor Microenvironment Represses T Cell Mitochondrial Biogenesis to Drive Intratumoral T Cell Metabolic Insufficiency and Dysfunction. Immunity (2016) 45(3):701–3. 10.1016/j.immuni.2016.08.009 27653602

[26] Chang CH Curtis JD Maggi LB Jr. Faubert B Villarino AV O’Sullivan D. Posttranscriptional Control of T Cell Effector Function by Aerobic Glycolysis. Cell (2013) 153(6):1239–51. 10.1016/j.cell.2013.05.016 PMC3804311 23746840

[27] Noman MZ Desantis G Janji B Hasmim M Karray S Dessen P. PD-L1 Is a Novel Direct Target of HIF-1alpha, and Its Blockade Under Hypoxia Enhanced MDSC-Mediated T Cell Activation. J Exp Med (2014) 211(5):781–90. 10.1084/jem.20131916 PMC4010891 24778419

[28] Nguyen LT Ohashi PS. Clinical Blockade of PD1 and LAG3–Potential Mechanisms of Action. Nat Rev Immunol (2015) 15(1):45–56. 10.1038/nri3790 25534622

[29] Ballbach M Dannert A Singh A Siegmund DM Handgretinger R Piali L. Expression of Checkpoint Molecules on Myeloid-Derived Suppressor Cells. Immunol Lett (2017) 192:1–6. 10.1016/j.imlet.2017.10.001 28987474

[30] Thakur A Lum LG. *In Situ* Immunization by Bispecific Antibody Targeted T Cell Therapy in Breast Cancer. Oncoimmunology (2016) 5(3):e1055061. 10.1080/2162402X.2015.1055061 27141330 PMC4839366

[31] Guedan S Posey AD Jr Shaw C Wing A Da T Patel PR. Enhancing CAR T Cell Persistence Through ICOS and 4-141BB Costimulation. JCI Insight (2018) 3(1):1–18. 10.1172/jci.insight.96976 PMC5821198 29321369

[32] Kawalekar OU OC RS Fraietta JA Guo L McGettigan SE Posey AD Jr.. Distinct Signaling of Coreceptors Regulates Specific Metabolism Pathways and Impacts Memory Development in CAR T Cells. Immunity (2016) 44(3):712. 10.1016/j.immuni.2016.02.023 28843072

[33] Priceman SJ Gerdts EA Tilakawardane D Kennewick KT Murad JP Park AK. Co-Stimulatory Signaling Determines Tumor Antigen Sensitivity and Persistence of CAR T Cells Targeting PSCA+ Metastatic Prostate Cancer. Oncoimmunology (2018) 7(2):e1380764. 10.1080/2162402X.2017.1380764 29308300 PMC5749625

[34] Zhao Z Condomines M van der Stegen SJC Perna F Kloss CC Gunset G. Structural Design of Engineered Costimulation Determines Tumor Rejection Kinetics and Persistence of CAR T Cells. Cancer Cell (2015) 28(4):415–28. 10.1016/j.ccell.2015.09.004 PMC5003056 26461090

[35] Goldberg MA Dunning SP Bunn HF. Regulation of the Erythropoietin Gene: Evidence That the Oxygen Sensor is a Heme Protein. Science (1988) 242(4884):1412–5. 10.1126/science.2849206 2849206

[36] Bache M Kappler M Said HM Staab A Vordermark D. Detection and Specific Targeting of Hypoxic Regions Within Solid Tumors: Current Preclinical and Clinical Strategies. Curr Med Chem (2008) 15(4):322–38. 10.2174/092986708783497391 18288988

[37] Brown JM Wilson WR. Exploiting Tumour Hypoxia in Cancer Treatment. Nat Rev Cancer (2004) 4(6):437–47. 10.1038/nrc1367 15170446

[38] Atkuri KR Herzenberg LA Niemi AK Cowan T Herzenberg LA. Importance of Culturing Primary Lymphocytes at Physiological Oxygen Levels. Proc Natl Acad Sci USA (2007) 104(11):4547–52. 10.1073/pnas.0611732104 PMC1838638 17360561

[39] Lum LG Thakur A Elhakiem A Alameer L Dinning E Huang M. Anti-CS1 × Anti-CD3 Bispecific Antibody (BiAb)-Armed Anti-CD3 Activated T Cells (CS1-BATs) Kill CS1^+^ Myeloma Cells and Release Type-1 Cytokines. Front Oncol (2020) 10:544. 10.3389/fonc.2020.00544 32432032 PMC7214537

[40] Zitron IM Thakur A Norkina O Barger GR Lum LG Mittal S. Targeting and Killing of Glioblastoma With Activated T Cells Armed With Bispecific Antibodies. BMC Cancer (2013) 13:83. (please see cytokine data on page 11). 10.1186/1471-2407-13-83 23433400 PMC3599512

[41] Ueda M Joshi ID Dan M Uberti JP Chou TH Sensenbrenner LL. Preclinical Studies for Adoptive Immunotherapy in Bone Marrow Transplantation: II. Generation of Anti-CD3 Activated Cytotoxic T Cells From Normal Donors and Autologous Bone Marrow Transplant Candidates. Transplantation (1993) 56:351–6. 10.1097/00007890-199308000-00019 8356589

[42] Uberti JP Joshi I Ueda M Martilotti F Sensenbrenner LL Lum LG. Preclinical Studies Using Immobilized OKT3 to Activate Human T Cells for Adoptive Immunotherapy: Optimal Conditions for the Proliferation and Induction of Non-MHC-Restricted Cytotoxicity. Clin Immunol Immunopathol (1994) 70:234–40. 10.1006/clin.1994.1034 8313660

[43] Curti BD Ochoa AC Powers GC Kopp WC Alvord WG Janik JE. Phase I Trial of Anti-CD3-Stimulated CD4^+^ T Cells, Infusional Interleukin-2, and Cyclophosphamide in Patients With Advanced Cancer. J Clin Oncol (1998) 16:2752–60. 10.1200/JCO.1998.16.8.2752 9704728

[44] Lum LG. Immunotherapy With Activated T Cells After High Dose Chemotherapy and PBSCT for Breast Cancer. Dicke KA Keating A, editors. Charlottesville, NY: Carden Jennings (2000) p. 95–105.

[45] Mata M Gerken C Nguyen P Krenciute G Spencer DM Gottschalk S. Inducible Activation of MyD88 and CD40 in CAR T Cells Results in Controllable and Potent Antitumor Activity in Preclinical Solid Tumor Models. Cancer Discov (2017) 7(11):1306–19. 10.1158/2159-8290.CD-17-0263 PMC5780189 28801306

