# Supplementary material for: Bispecific Antibody Armed Metabolically Enhanced Headless CAR T Cells
Source: Front Immunol. 2021 Jul 5;12:690437. doi: 10.3389/fimmu.2021.690437 (PMC8288104; doi:10.3389/fimmu.2021.690437)
Supplement: Supplementary file 1 [file DataSheet_1.pdf]

Figure Legends

Figure S1. Serial or Repeated Cytotoxicity Mediated by hCART Transduced with Different Vectors. Specific cytotoxicity up to 4 rounds of serial killing are shown for unarmed (without BiAb loading) hCART$_{41BB\zeta}$, hCART $_{ICOS\zeta}$, hCART $_{ICOS\text{-}27\zeta}$, COATC, ATC (indicated by dashed lines) and for armed HER2 hCART $_{41BB\zeta}$, HER2 hCART$_{ICOS\zeta}$, HER2 hCART$_{ICOS\text{-}27\zeta}$, HER2 COACT, HER2 BATs (indicated by solid lines) against MCF-7 cell line. Each killing was monitored up to 96 hours by RTCA at E:T of 10:1.

Figure S2. Effect of Hypoxia on Effector Cell Functions. Shows dose titration of hypoxia inducing cobalt chloride ($CoCl_2$) on the viability of MCF-7 target cell line (**upper panel**) and effector T cells (**lower panel**). Cytotoxic effects of hypoxia were measured by RTCA in MCF-7 cell line; apoptotic effect of hypoxia on effector populations was measured by flow cytometry. Both, MCF-7 cells and effector T cells showed < 10% death at 100 μM $CoCl_2$, therefore, 100 μM dose of $CoCl_2$ was used in experiments measuring the effect of hypoxia on effector cell functions of bionic T cells.

Figure S3. hCART$_{41BB\zeta}$ Show Metabolic Fitness. Shows metabolic reprogramming on day zero or day seven of hCART expressing either 28ζ or 41BBζ (BBζ) intracellular domains. hCART$_{41BB\zeta}$ show increased spare respiratory capacity, which is associated with enhanced survival and persistence under stress. Spare respiratory capacity was measured by a sea horse assay. OCR: Oxygen consumption rate.

Sequence of 4-1BBζ, ICOSζ and ICOS-27ζ Constructs

CD8 Leader
Flag tag
CD8 Hinge
CD8 TM
ICOS TM
4-1BB ICD
ICOS ICD
CD27 ICD
CD3z ICD
TM = Transmembrane
ICD = Intra Cellular Domain

Sequence of 4-1BBζ Construct
MALPVTALLLPLALLLHAARPDYKDHDGDYKDHDIDYKDDDDKGSTTTPAPRPPTPAP
TIASQPLSLRPEACRPAAGGAVHTRGLDFACDIYIWAPLAGTCGVLLLSLVITLYCSAKR
GRKKLLYIFKQPFMRPVQTTQEEDGCSCRFPEEEEGGCELRVKFSRSADAPAYKQGQNQ
LYNELNLGRREEYDVLDKRRGRDPEMGGKPRRKNPQEGLYNELQKDKMAEAYSEIGM
KGERRRGKGHDGLYQGLSTATKDTYDALHMQALPPR

Sequence of ICOSζ Construct
MALPVTALLLPLALLLHAARPDYKDHDGDYKDHDIDYKDDDDKGSTTTPAPRPPTPAP
TIASQPLSLRPEACRPAAGGAVHTRGLDFACDIWLPIGCAAFVVVCILGCILICWLTKKK
YSSSVHDPNGEYMFMRAVNTAKKSRLTDVTLTSRVKFSRSADAPAYKQGQNQLYNEL
NLGRREEYDVLDKRRGRDPEMGGKPRRKNPQEGLYNELQKDKMAEAYSEIGMKGERR
RGKGHDGLYQGLSTATKDTYDALHMQALPPR

Sequence of ICOS-27ζ Construct
MALPVTALLLPLALLLHAARPDYKDHDGDYKDHDIDYKDDDDKGSTTTPAPRPPTPAP
TIASQPLSLRPEACRPAAGGAVHTRGLDFACDIWLPIGCAAFVVVCILGCILICWLTKKK
YSSSVHDPNGEYMFMRAVNTAKKSRLTDVTLQRRKYRSNKGESPVEPAEPCRYSCPRE
EEGSTIPIQEDYRKPEPACSPLRVKFSRSADAPAYKQGQNQLYNELNLGRREEYDVLDK
RRGRDPEMGGKPRRKNPQEGLYNELQKDKMAEAYSEIGMKGERRRGKGHDGLYQGLS
TATKDTYDALHMQALPPR

Table S1. Tukey's multiple comparisons test

	COATC		hCART$_{41BB\zeta}$		hCART$_{ICOS\zeta}$		hCART$_{icos\text{-}27\zeta}$		BATs	
	Summary	p value	Summary	p value	Summary	p value	Summary	p value	Summary	p value
**IL-2**										
UA vs. HER2Bi	ns	0.1827	ns	0.4807	ns	0.677	*	0.0105	ns	0.7816
UA vs. EGFRBi	ns	0.5015	ns	0.4319	*	0.0188	****	<0.0001	ns	0.5454
HER2Bi vs. EGFRBi	*	0.0306	ns	0.9952	ns	0.0716	****	<0.0001	ns	0.9131
**IFN-γ**										
UA vs. HER2Bi	ns	0.3552	*	0.0187	ns	0.3877	*	0.0179	ns	0.1066
UA vs. EGFRBi	ns	0.0582	*	0.0371	ns	0.4841	*	0.0237	ns	0.0973
HER2Bi vs. EGFRBi	ns	0.4619	ns	0.8989	ns	0.9805	ns	0.9818	ns	0.9981
**TNF-α**										
UA vs. HER2Bi	ns	0.5581	**	0.0014	*	0.0252	**	0.0018	*	0.0498
UA vs. EGFRBi	**	0.0037	**	0.0018	ns	0.0515	****	<0.0001	ns	0.054
HER2Bi vs. EGFRBi	*	0.0178	ns	0.977	ns	0.8922	**	0.0028	ns	0.9986

Table S2. Tukey's multiple comparisons test

	COATC		hCART$_{41BB\zeta}$		hCART$_{ICOS\zeta}$		hCART$_{icos\text{-}27\zeta}$		BATs	
**MIP-1β**										
	Summary	p value	Summary	p value	Summary	p value	Summary	p value	Summary	p value
UA vs. HER2Bi	ns	0.1586	ns	0.1503	*	0.0328	*	0.0165	***	0.0008
UA vs. EGFRBi	*	0.0385	**	0.0048	*	0.0408	*	0.0248	**	0.0021
HER2Bi vs. EGFRBi	ns	0.6409	ns	0.112	ns	0.9892	ns	0.9624	ns	0.7411
**IP-10**										
UA vs. HER2Bi	ns	0.1098	**	0.0081	ns	0.3807	**	0.008	ns	0.3959
UA vs. EGFRBi	ns	0.7556	****	<0.0001	ns	0.1966	*	0.0119	ns	0.3807
HER2Bi vs. EGFRBi	ns	0.311	****	<0.0001	ns	0.8776	ns	0.961	ns	0.9994
**RANTES**										
UA vs. HER2Bi	ns	0.2323	ns	0.0784	ns	0.0764	ns	0.0572	ns	0.1345
UA vs. EGFRBi	*	0.0265	****	<0.0001	**	0.0022	**	0.0095	ns	0.3389
HER2Bi vs. EGFRBi	ns	0.3684	****	<0.0001	ns	0.0935	ns	0.499	ns	0.7936

Figure S1

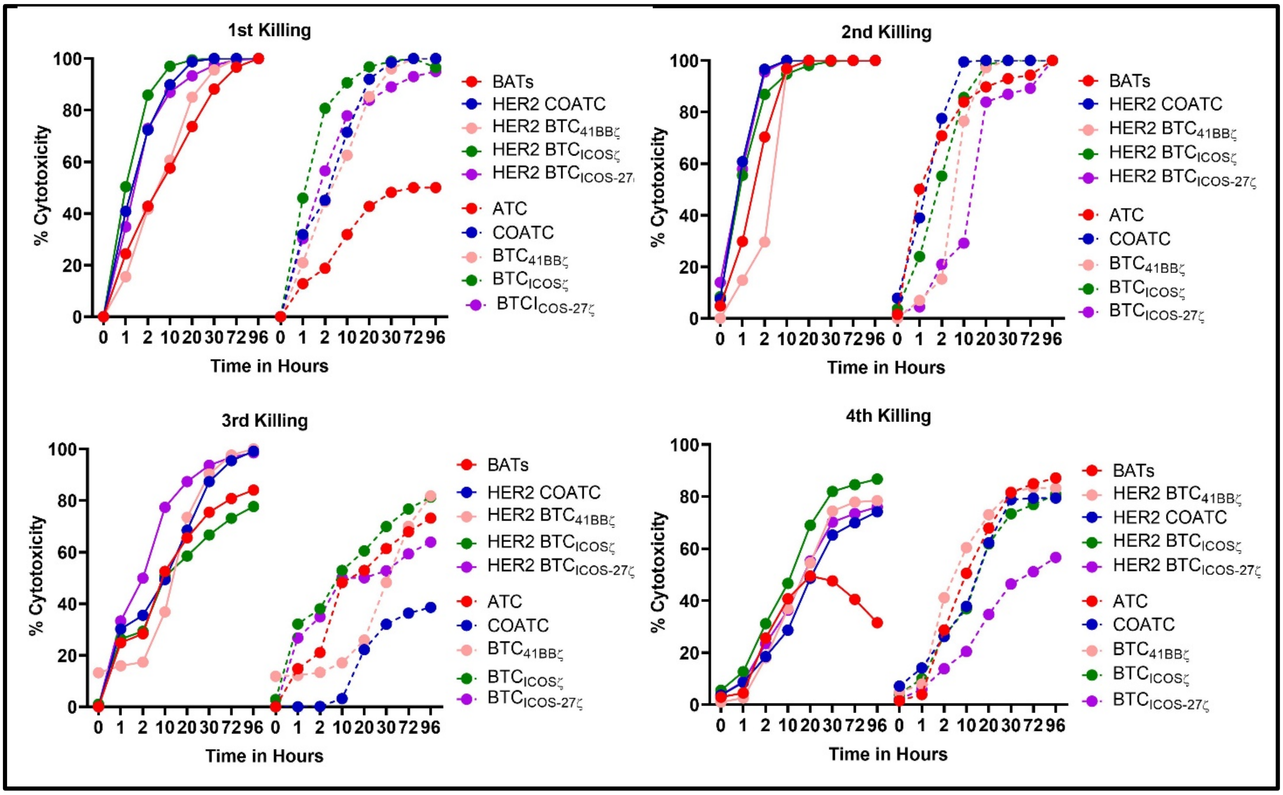
1st Killing
2nd Killing
3rd Killing
4th Killing
% Cytotoxicity
Time in Hours
BATs
HER2 COATC
HER2 BTC41BBζ
HER2 BTCICOSζ
HER2 BTCICOS-27ζ
ATC
COATC
BTC41BBζ
BTCICOSζ
BTCICOS-27ζ

Figure S2

Effect of $CoCl_2$ Induced Hypoxia on Tumor Cell Line MCF-7

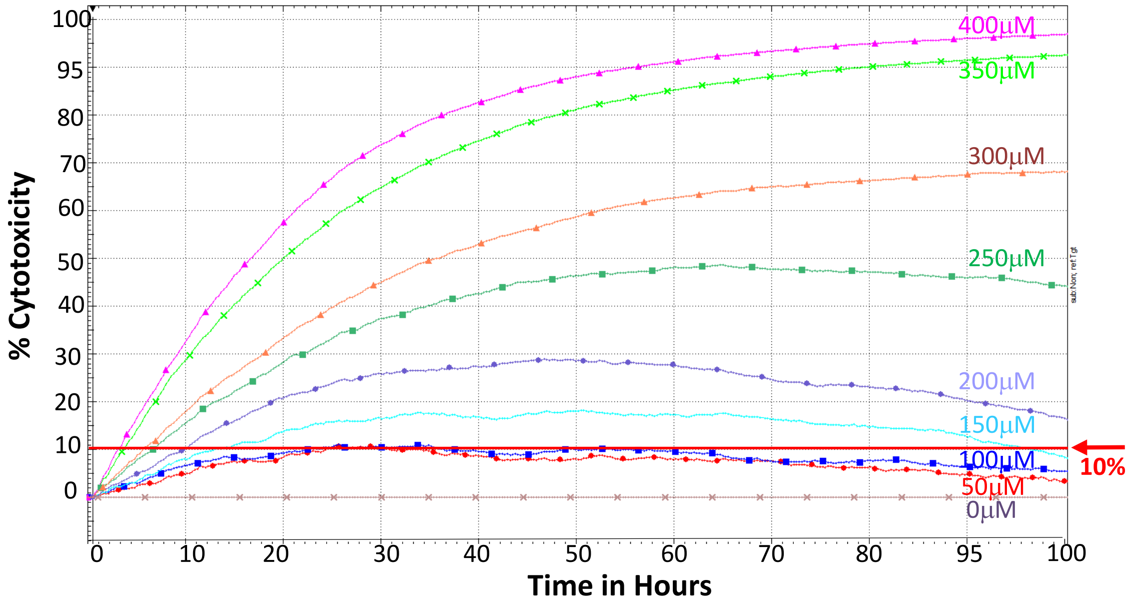

Effect of $CoCl_2$ Induced Hypoxia on T cells

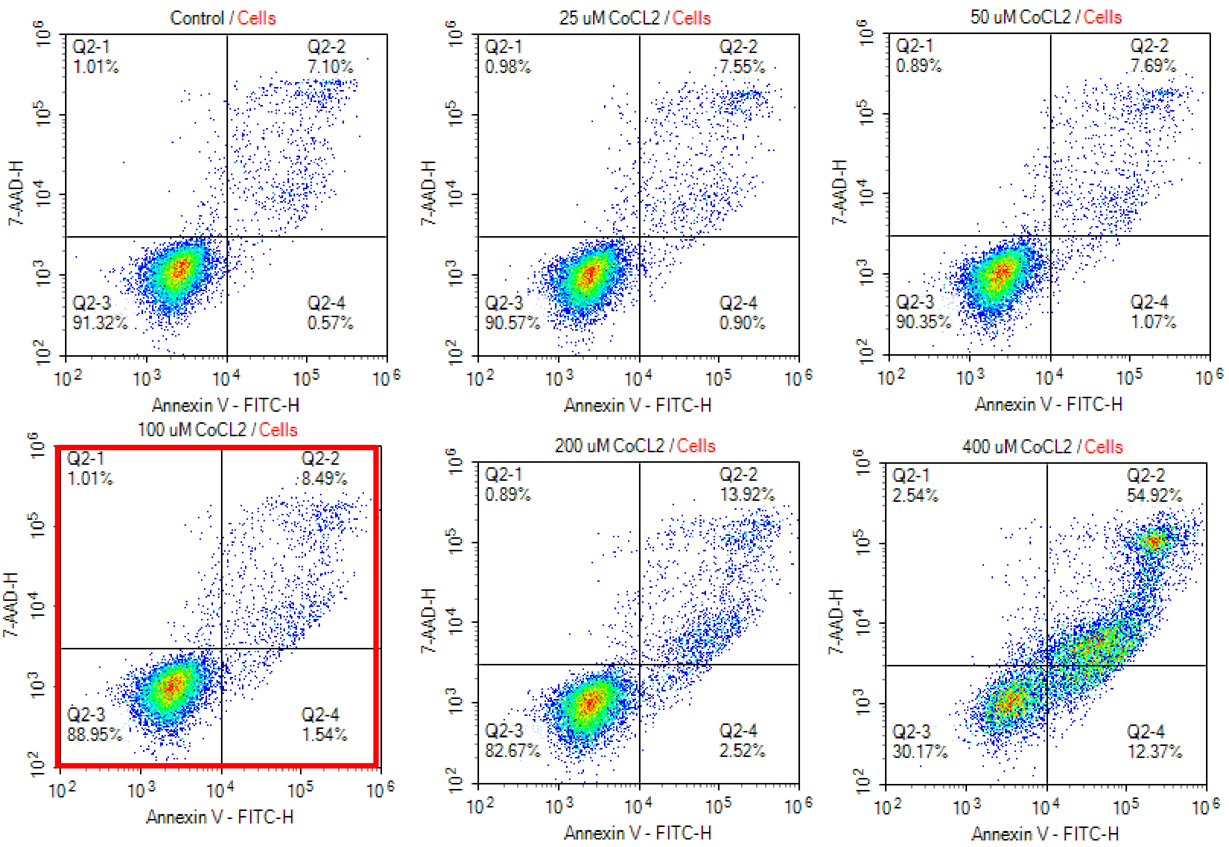

Figure S3

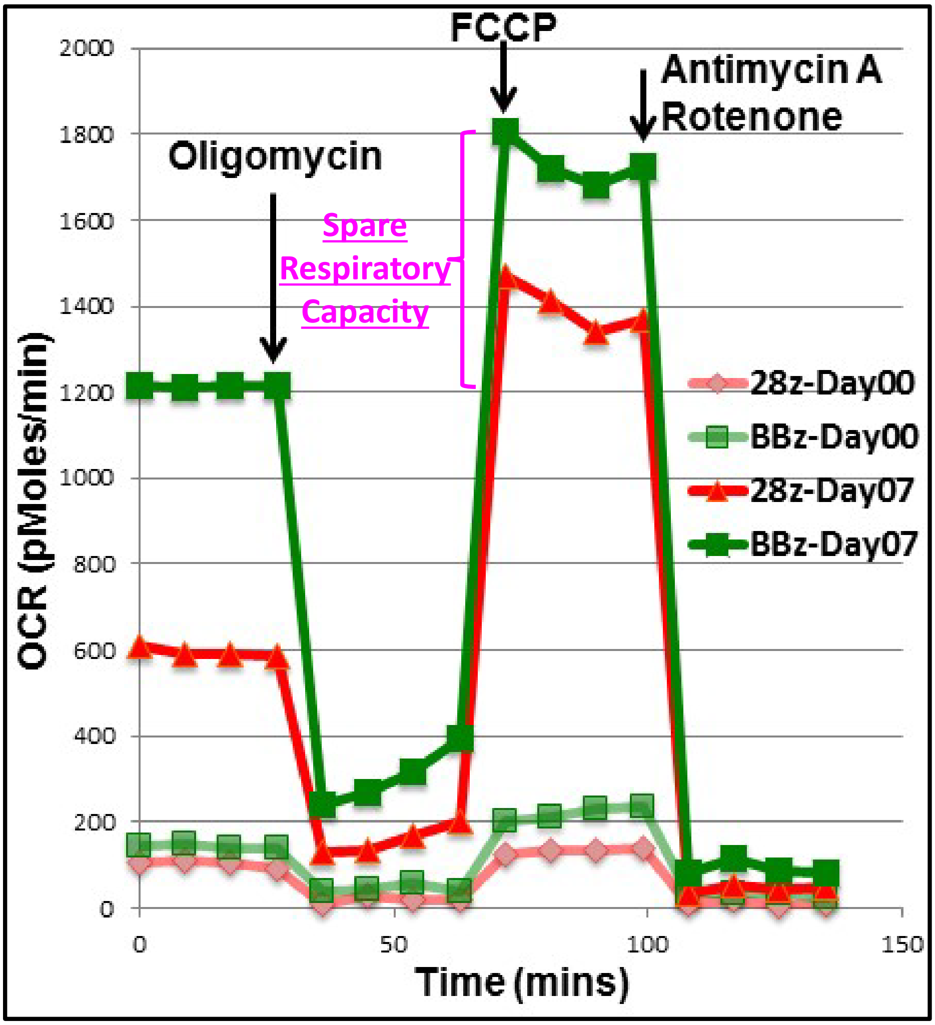

FCCP
Antimycin A
Rotenone
Oligomycin
Spare
Respiratory
Capacity
OCR (pMoles/min)
2000
1800
1600
1400
1200
1000
800
600
400
200
0
0
50
100
150
Time (mins)
28z-Day00
BBz-Day00
28z-Day07
BBz-Day07
